# A polymorphic tetranucleotide repeat in the *CYP19* gene and male breast cancer

**DOI:** 10.1054/bjoc.1999.1085

**Published:** 2000-03-06

**Authors:** I E Young, K M Kurian, M A F MacKenzie, I H Kunkler, B B Cohen, M L Hooper, A H Wyllie, C M Steel

**Affiliations:** Sir Alastair Currie CRC Laboratories, University of Edinburgh, Department of Pathology, Molecular Medicine Centre and Department of Clinical Oncology, Western General Hospital, Crewe Road, Edinburgh, EH4 2XU, UK; School of Biomedical Sciences, Bute Medical Building, University of St Andrews, St Andrews, Fife, KY16 9TS, UK; Department of Pathology, University of Cambridge, Tennis Court Road, Cambridge, CB2 1QP, UK

**Keywords:** CYP19, male breast cancer

## Abstract

The *CYP19* gene codes for the aromatase enzyme that is involved in the synthesis of oestrogens. This case–control study examines the relationship between a tetranucleotide repeat sequence in the *CYP19* gene and the development of male breast cancer. No significant differences were found between male breast cancer cases and controls. © 2000 Cancer Research Campaign


*British Journal of Cancer* (2000) **82**(7), 1247–1248
© 2000 Cancer Research Campaign
DOI: 10.1054/ bjoc.1999.1085, available online at http://www.idealibrary.com on IDEAL®

### A polymorphic tetranucleotide repeat in the *CYP19* gene and male breast cancer

**IE Young[1], KM Kurian[1], MAF MacKenzie[1], IH Kunkler[2], BB Cohen[3], ML Hooper[1], AH Wyllie[1,4] and CM Steel[3]**

[1]Sir Alastair Currie CRC Laboratories, University of Edinburgh, Department of Pathology, Molecular Medicine Centre and [2]Department of Clinical Oncology, Western General Hospital, Crewe Road, Edinburgh EH4 2XU, UK; [3]School of Biomedical Sciences, Bute Medical Building, University of St Andrews, St Andrews, Fife KY16 9TS, UK; [4]Department of Pathology, University of Cambridge, Tennis Court Road, Cambridge CB2 1QP, UK

**Summary** The *CYP19* gene codes for the aromatase enzyme that is involved in the synthesis of oestrogens. This case–control study examines the relationship between a tetranucleotide repeat sequence in the *CYP19* gene and the development of male breast cancer. No significant differences were found between male breast cancer cases and controls. © 2000 Cancer Research Campaign

**Keywords**: CYP19; male breast cancer

The *CYP19* gene (located on chromosome 15q21.1) codes for the aromatase enzyme that controls the rate-limiting step in the pathway of oestrogen synthesis from steroid precursors. It is known that an increased risk of breast cancer in males is associated with elevated serum oestrogen levels, for example in Klinefelter's syndrome (Jackson et al, 1965). The aromatase enzyme has been observed within the stromal cells in a greater proportion of male breast carcinomas than gynaecomastia cases, suggesting that locally produced oestrogens may also have a significant role in the development of male breast cancer (Sasano et al, 1996). It is therefore possible that variation in expression of the *CYP19* gene could affect the risk of developing male breast cancer.

A polymorphic tetranucleotide (TTTA) repeat sequence is found in intron 5 of the *CYP19* gene, 79 nucleotides downstream from exon 4 (Means et al, 1989). This repeat sequence is relatively close to the exon–intron border and may therefore be involved in the determination of splicing sites (Kristensen et al, 1998). The aim of this study was to determine whether the development of male breast cancer is influenced by the length of this tetranucleotide repeat sequence in the *CYP19* gene.

### METHODS

### Case and control population selection

Male cases were taken from a consecutive series of 76 male breast cancer patients treated in the South East of Scotland between 1974 and 1998. Samples were available for DNA extraction in 64 of these cases. Control DNA samples were obtained from 79 healthy males representative of the South East Scotland population. Further details of male breast cancer cases and controls have previously been described (Young et al, 1999). Ethical approval for the study was obtained through the Lothian Regional Ethics Committee.

### Laboratory methods

DNA extraction was carried out from whole blood by standard phenol–chloroform extraction. DNA extraction from wax-embedded tissue was from 10-μm sections incubated at 55°C with a lysis buffer and proteinase K.

Primers as previously described (Polymeropoulos et al, 1991) were used for polymerase chain reaction (PCR): CYP19-F: 5′-GCAGGTACTTAGTTAGCTAC-3′; CYP19-R: 5′-TTACAGT-GAGCCAAGGTCGT-3′. These generate PCR fragments that include the polymorphic site. PCR reactions were performed in 50-μl aliquots, each containing 1 × buffer, 2 mM magnesium chloride ($MgCl_2$), 200 μM deoxynucleoside triphosphates, 40 pmol of each primer, 1 unit of *Taq* polymerase (Life Technologies™) and approximately 100 ng DNA. The amplification was performed using an OmniGene thermal cycler (Hybaid, UK) under the following conditions: initial denaturation at 94°C for 3 min; amplification for 38 cycles, with denaturation at 94°C for 45 s, annealing at 53°C for 45 s and extension at 72°C for 45 s; final extension at 72°C for 10 min.

The products were denatured and then run on 6% polyacrylamide gels with a 10 bp DNA ladder. The number of TTTA repeats in homozygous products were measured by cycle sequencing and these were used as size standards.

### Data analysis

The distribution of alleles of the *CYP19* gene, comparing male breast cancer patients with controls, was analysed using the Mann–Whitney test. Odds ratios with 95% confidence intervals were calculated to show the risk of developing male breast cancer associated with each allele.

*Received 1 June 1999*
*Revised 7 November 1999*
*Accepted 22 November 1999*

*Correspondence to:* IE Young, Department of General Surgery, Dumfries & Galloway Royal Infirmary, Bankend Road, Dumfries DG1 4AP, UK

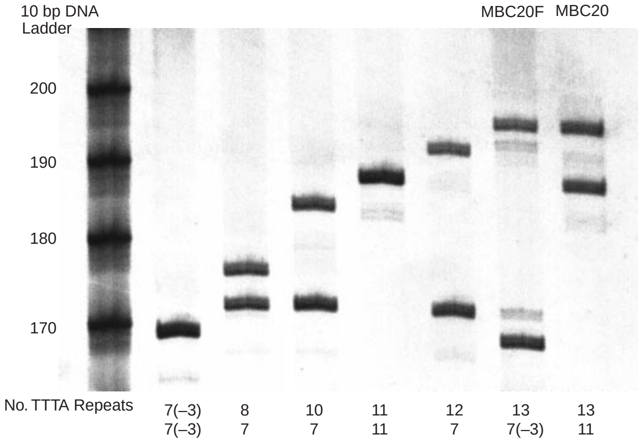

**Figure 1** Examples of the seven different alleles of the *CYP19* gene found in male breast cancer patients and controls

**Table 1** Distribution of alleles of the *CYP19* gene in male breast cancer patients and controls

| Allele | PCR product length (bp) | Cases ($n$ = 108) | Controls ($n$ = 158) | Odds ratio & 95% confidence interval |
|---|---|---|---|---|
| $(TTTA)_{7-3}$ | 168 | 35 (32.4%) | 48 (30.4%) | 1.10 (0.649–1.86) |
| $(TTTA)_{7}$ | 171 | 20 (18.5%) | 30 (19.0%) | 0.970 (0.518–1.82) |
| $(TTTA)_{8}$ | 175 | 11 (10.2%) | 16 (10.1%) | 1.01 (0.448–2.26) |
| $(TTTA)_{10}$ | 183 | 1 (0.93%) | 2 (1.3%) | 0.729 (0.0653–8.14) |
| $(TTTA)_{11}$ | 187 | 36 (33.3%) | 57 (36.1%) | 0.886 (0.529–1.48) |
| $(TTTA)_{12}$ | 191 | 4 (3.7%) | 5 (3.2%) | 1.18 (0.309–4.49) |
| $(TTTA)_{13}$ | 195 | 1 (0.93%) | 0 | – |
| Heterozygosity | | 79.6% | 79.7% | |

### RESULTS

PCR was unsuccessful with ten of the DNA samples derived from archival wax-embedded tissue sections, giving a total of 54 case samples (108 alleles) analysed.

Seven different alleles of the *CYP19* gene were detected (Figure 1). The allele distribution of the *CYP19* gene in male breast cancer patients and controls is shown in Table 1. There were no significant differences in the distribution of alleles between cases and controls ($P$ = 0.838). We have found two different alleles containing seven TTTA repeats (corresponding to PCR products of 168 bp and 171 bp in length). Cycle sequencing of these alleles revealed that the shorter allele, designated $(TTTA)_{7-3}$, had a 3-bp (TTC) deletion 50 bp upstream from the 5′ end of the TTTA repeat sequence. One of the male breast cancer patients (MBC20) had an allele containing 13 repeats. A blood sample was obtained from his father (MBC20F), who had not had breast cancer. Analysis of DNA extracted from this showed the same $(TTTA)_{13}$ allele.

### DISCUSSION

This is the first study that attempts to determine whether the development of male breast cancer is influenced by the length of the tetranucleotide repeat sequence within intron 5 of the *CYP19* gene. Two recent studies have determined the distribution of alleles among female breast cancer patients and controls. The first of these studies (Kristensen et al, 1998) found five different alleles containing 7, 8, 9, 11 and 12 repeats. The allele containing 12 repeats was found significantly more frequently in female breast cancer patients than in controls. The second of the studies (Siegelmann-Danieli and Buetow, 1999) described eight different alleles by PCR product length. Details of TTTA repeat number were not given. Alleles of 168 bp and 171 bp in length were found, presumably corresponding to the two different alleles containing seven repeats found in our study, although the 3-bp deletion was not characterized. This deletion has, however, been described previously in a Japanese study (Kurosaki et al, 1997). From their study, Siegelmann-Danieli and Buetow (1999) conclude that the 171 bp allele represents a high-risk allele, whereas the 187 bp and 191 bp alleles (corresponding to 11 and 12 repeats respectively, from our data) are considered to confer low risk. These conclusions are contradictory to those drawn by Kristensen et al (1998).

Contrary to published (and mutually incompatible) findings in female breast cancer, our study found no significant differences in distribution of alleles between male breast cancer cases and controls. Patient MBC20, who has an allele with 13 repeats, was diagnosed with breast cancer at a very young age (26 years), but has no family history of cancer. This certainly represents a rare variant within the South East Scotland population, but is most likely to be an incidental finding.

### ACKNOWLEDGEMENTS

We thank the following: Mr R Morris and Dr S Bader for technical advice; Dr T Anderson, Dr A McGregor, Dr I Nawroz, Dr K Ramesar and Dr AM Lutfy for making available the archival wax-embedded tissue sections; Miss G Kerr and the medical records staff in the department of Clinical Oncology, Western General Hospital, Edinburgh; Staff in the Department of Blood Transfusion Medicine, Royal Infirmary of Edinburgh and in the Department of ENT Surgery, City Hospital, Edinburgh for providing some of the control samples. This work has been funded by grants from the Royal College of Surgeons of Edinburgh, the Sarah Percy Fund, the Melville Trust for the Care and Cure of Cancer and the Robertson Trust.

### REFERENCES

Jackson AW, Muldal S, Ockey CH and O'Connor PJ (1965) Carcinoma of the male breast in association with the Klinefelter syndrome. *Br Med J* **1**: 223–225

Kristensen VN, Andersen TI, Lindblom A, Erikstein B, Magnus P and Børresen-Dale A-L (1998) A rare CYP19 (aromatase) variant may increase the risk of breast cancer. *Pharmacogenetics* **8**: 43–48

Kurosaki K, Saitoh H, Oota H, Watanabe Y, Kiuchi M and Ueda S (1997) Combined polymorphism associated with a 3-bp deletion in the 5′-flanking region of a tetrameric short tandem repeat at the CYP19 locus. *Nippon Hoigaku Zasshi* **51**: 191–195

Means GD, Mahendroo MS, Corbin CJ, Mathis JM, Powell FE, Mendelson CR and Simpson ER (1989) Structural analysis of the gene encoding human aromatase cytochrome P-450, the enzyme responsible for estrogen biosynthesis. *J Biol Chem* **264**: 19385–19389

Polymeropoulos MH, Xiao H, Rath DS and Merril CR (1991) Tetranucleotide repeat polymorphism at the human aromatase cytochrome P-450 gene (CYP19). *Nucleic Acids Res* **19**: 195

Sasano H, Kimura M, Shizawa S, Kimura N and Nagura H (1996) Aromatase and steroid receptors in gynaecomastia and male breast carcinoma: an immunohistochemical study. *J Clin Endocrinol Metab* **81**: 3063–3067

Siegelmann-Danieli and Buetow KH (1999) Constitutional genetic variation at the human aromatase gene (Cyp19) and breast cancer risk. *Br J Cancer* **79**: 456–463

Young IE, Kurian KM, Annink C, Kunkler IH, Anderson VA, Cohen BB, Hooper ML, Wyllie AH and Steel CM (1999) A polymorphism in the *CYP17* gene is associated with male breast cancer. *Br J Cancer* **81**: 141–143

 *© 2000 Cancer Research Campaign*
